# Functional Impairment and Painful Physical Symptoms in Patients with Major Depressive Disorder Treated with Antidepressants: Real-World Evidence from the Middle East

**DOI:** 10.2174/1745017901713010145

**Published:** 2017-09-30

**Authors:** Jihyung Hong, Diego Novick, Maria Victoria Moneta, Ahmed El-Shafei, Héctor Dueñas, Josep Maria Haro

**Affiliations:** 1 Department of Healthcare Management, Gachon University, Seongnam, South Korea; 2 Eli Lilly and Company, Windlesham, Surrey, United Kingdom; 3 Parc Sanitari Sant Joan de Déu, Fundació Sant Joan de Déu, CIBERSAM, Universitat de Barcelona, Barcelona, Spain;; 4 Eli Lilly (Suisse) S.A., Dubai, United Arab Emirates; 5 Eli Lilly de Mexico, Mexico City

**Keywords:** Depression, Antidepressant, Duloxetine, SSRI, Functioning, Middle East

## Abstract

**Background:**

The Global Burden of Disease 2010 study reported the relative size of major depressive disorder (MDD) burden to be greater in the Middle East and North Africa than anywhere else. However, little research has been carried out to examine the comparative effectiveness of antidepressants in this region.

**Objective:**

To assess and compare functioning levels in Middle Eastern patients with MDD treated with either duloxetine or a selective serotonin reuptake inhibitor (SSRI), and to examine the impacts of depression-related pain on functioning by the type of treatment.

**Method:**

This post-hoc analysis, which focused on Middle Eastern patients, used data from a 6-month prospective observational study that included 1,549 MDD patients without sexual dysfunction. Levels of functional impairment and depression-related pain were assessed using the Sheehan Disability Scale (SDS) and the modified Somatic Symptom Inventory, respectively. A mixed model with repeated measures (MMRM) was employed.

**Results:**

The mean age of the patients was 37.3 (SD=8.4) years, and 34.6% were female. Patient functioning was, on average, moderately impaired at baseline, but improved substantially during follow-up in both the duloxetine (n=152) and the SSRI (n=123) cohorts. The MMRM results showed a lower level of functional impairment at 24 weeks in the duloxetine cohort than in the SSRI cohort (p<0.001). Pain severity at baseline was positively associated with functional impairment during follow-up only in the SSRI cohort (p=0.003).

**Conclusion:**

Duloxetine-treated MDD patients achieved better functioning than SSRI-treated patients. This treatment difference was partly driven by depression-related pain.

## 1 INTRODUCTION

Depression is one of the most prevalent, disabling and costly mental illnesses, currently affecting over 300 million people worldwide [1]. According to the World Mental Health (WHM) survey conducted in 18 countries, the life-time prevalence of major depressive episode (MDE) was found to be up to 21%. The survey also revealed a great variation in the prevalence of MDE across different countries – it tended to be higher in some Western countries but lower in Asian and Middle Eastern countries. For instance, it was found to be 19.2% in the United States and 21.0% in France but 6.5% in China, 6.6% in Japan and 10.9% in Lebanon - the only Arab country included in the survey [2]. However, these estimates are based on data from 2001-2007, and emerging evidence suggests that the prevalence of depression in these latter countries is probably higher than what is reported in the survey. For example, the 2010 Global Burden of Disease (GBD) study reported the relative size of major depressive disorder (MDD) burden, measured in disability adjusted life years (DALYs), to be greater in the Middle East and North Africa (MENA) than anywhere else, particularly in women in this region [3, 4]. Unfortunately, this high level of MDD burden is expected to continue because wars and conflicts in the MENA region are likely to further increase the prevalence and the burden of MDD [5].

The high level of DALYs due to depression, which accounts for nearly half (40.5%) of the total DALYs associated with mental and substance use disorders [6], translates into a substantial economic burden of the disease. Although such estimates are not available in the MENA region, the total costs of depression were estimated to be about ¥2 trillion (about US$20 billion) in Japan (2005 value) [7], over US$6 billion in China (2002 value) [8], and over US$4 billion in South Korea (2005 value) [9]. Notably, indirect costs associated with lost productivity far exceeded direct medical costs of depression in all studies. This implies that patients with depression have substantial impairment in functioning and health-related quality of life (HRQoL).

Indeed, nearly two-thirds of MDD patients were reported to have severe impairment in functioning and HRQoL, respectively [10, 11]. Such impairment may be even more pronounced in countries, for instance those of the MENA region, where access to mental health care is severely limited due to the strong stigma attached to mental illness [12]. Furthermore, a high proportion of psychiatric patients also tend to somatise their emotional problems and communicate them in the form of physical symptoms in such cultures [13]. Many of them seek help first from primary care physicians and present to them with physical complaints such as headache, heart/chest pain, back pain, and abdominal pain, rather than psychological complaints. These physical symptoms, either as a manifestation of MDD or as a coexisting condition, not only delay the diagnosis and treatment of MDD but also further interfere with daily functioning and HRQoL in patients with MDD [14, 15].

Duloxetine hydrochloride is a potent and relatively balanced inhibitor of serotonin and norepinephrine reuptake (SNRI) [16]. It has been approved not only for the management of MDD and generalised anxiety disorder but also for pain-related conditions (chronic musculoskeletal pain, fibromyalgia, and diabetic peripheral neuropathic pain) in the United States. It has also been approved for some or all of them in many other countries, including the countries of the MENA region. Limited evidence, mostly from the United States and Europe, suggests that the dual action of SNRIs may more effectively control the symptoms of depression than those antidepressants inhibiting only one monoamine at least for the subgroup of MDD patients with concomitant pain [17-19]. This is because the pathophysiology of both conditions likely involve an imbalance of serotonin and norepinephrine [20]. More research is needed to establish this hypothesis, especially in the MENA region, where the effects of such agents have never been evaluated [21].

This *post-hoc* study aimed to assess and compare the level of functional impairment over time in MDD patients treated with either duloxetine or a selective serotonin reuptake inhibitor (SSRI) for up to 6 months in actual clinical settings in Saudi Arabia and the United Arab Emirates (UAE), using data from an international prospective observational study. This study also examined whether the impacts of depression-related pain on functioning varied with the type of treatment.

## 2 MATERIAL AND METHODS

### 2.1 Study Design

This study used data from a 6-month, multi-national, prospective, non-interventional, observational study. It was primarily designed to examine treatment-emergent sexual dysfunction (TESD) and other treatment outcomes among patients with MDD who were treated with either an SNRI or a selective serotonin reuptake inhibitor (SSRI) in actual clinical practice. A total of 1,549 patients, out of 1,647 patients who were enrolled between Nov 2007 and Nov 2008, were classified as “sexually active patients without sexual dysfunction at study entry”. This *post-hoc* study focused only on those patients from the Middle East (n=314) (Saudi Arabia [*n*=179] and United Arab Emirates (UAE) [*n*=135]).

This study followed the ethical standards of responsible local committees and the regulations of the participating countries. Ethical review board (ERB) approval was obtained as required by local laws. All patients provided informed consent for the provision and collection of the data. This study was also conducted in accordance with the ethical principles that have their origin in the Declaration of Helsinki and that are consistent with Good Pharmacoepidemology Practices (GPPs), as well as applicable laws and regulations of the countries where the study was conducted, as appropriate.

Further details of the study design have been published elsewhere [22-26].

### 2.2 Study Sample

Patients were eligible to participate in the study if they met the following inclusion criteria: (1) at least 18 years of age; (2) presenting with an episode of MDD in an outpatient setting, with MDD diagnosed according to the *International Statistical Classification of Diseases 10^th^ revision* (ICD-10) [27]or *Diagnostic and Statistical Manual of Mental Disorders-4th edition text revision* (DSM-IV-TR) [28] criteria; (3) the Clinical Global Impression-Severity (CGI-S) score of ≥4 (*i.e.*, at least moderately ill) [29]; (4) initiating or switching to any available SSRI or SNRI antidepressant in accordance with a treating psychiatrist’s discretion; (5) being sexually active without sexual dysfunction, as defined by Arizona Sexual Experience Scale (ASEX) [30]; and (6) providing consent to release data. The study excluded patients who (1) were participating in another study; (2) had a history of treatment-resistant depression; (3) had a past or current diagnosis of schizophrenia, schizophreniform or schizoaffective disorder, bipolar disorder, dysthymia, mental retardation or dementia; or (4) received any antidepressant within 1 week (1 month for fluoxetine) prior to study entry, with the exception of patients receiving an ineffective treatment for whom the immediate switch to an SSRI or an SNRI antidepressant was considered to be the best treatment option.

Patients were, however, not required to continue their initial medications. Changes in medication and dosing were possible at any time during follow-up, as determined by their treating psychiatrist.

### 2.3 Data Collection and Outcome Assessment

Data were collected during visits within the normal course of care. Subsequent data collection was targeted at week 8, week 16, and week 24 from the baseline visit (*i.e.*, study entry). Patient socio-demographics and clinical history were recorded at the baseline assessment. Clinical severity of depression was assessed at each visit using the CGI-S [29] and the 16-item Quick Inventory of Depressive Symptomatology Self-Report (QIDS-SR_16_) [31].

Functional impairment was also assessed at each visit using the Sheehan Disability Scale (SDS) [32]. It is a brief self-report inventory that assesses functional impairment in work/school, social life, and family life. The level of functional impairment in each of the three domains is rated from 0 to 10. The level of global functional impairment is determined with the sum of the three subscores. A higher score indicates a higher level of functional impairment.

Depression-related pain severity was measured using the pain-related items of the Somatic Symptom Inventory (SSI), which includes headaches, heart/chest pain, neck pain, abdominal pain, lower back pain, joint pain, and muscular soreness [33]. PPS status was also assessed as painful physical symptom negative (PPS-) or positive (PPS+); PPS+ was defined as a mean score of at least 2 for the seven pain-related items of the SSI.

### 2.4 Statistical Analysis

This *post-hoc* study included 275 patients, out of the 314 patients from Saudi Arabia and UAE, who (1) initiated either duloxetine or an SSRI as monotherapy at baseline, and (2) who had non-missing data on the QIDS-SR_16_ score at baseline with at least one non-missing QIDS-SR_16_ score during follow-up (n=152 in the duloxetine cohort and n=123 in the SSRI cohort). This study analysed the patient observations up to the point where their initial medications were discontinued (n=127 [83.6%] in the duloxetine cohort and n=97 [78.9%] in the SSRI cohort available at 24 weeks).

The baseline socio-demographic and clinical characteristics of the two treatment cohorts were summarised and compared using the Chi-square test (for categorical variables) and the Mann-Whitney test (for continuous variables). The level of functional impairment (both SDS total scores and SDS subscores) at each visit was also described and compared using the Mann-Whitney test. The treatment difference in functional impairment at each visit was also expressed in terms of the effect size, using Cohen’s d [34].

The mixed effects models for repeated measures (MMRM) analysis was used to estimate and compare the levels of functional impairment (SDS total, work, social life, and family life) during follow-up between the two treatment groups. The models were adjusted for age, gender, SSI-pain score at baseline, QIDS-SR_16_ score at baseline, the baseline value of the outcome modelled, and visit number. The models were further adjusted for the following variables if they appeared to be significant (p<0.1) when each one of them was added to the above model: independent living (living in his/her own house), living with a spouse/partner, employment status, having MDD episodes in the 24 months prior to study entry, MDD hospitalisations in the 24 months prior to study entry, and the number of any significant pre-existing comorbidities. Finally, the models also included the interaction term between time (visit number) and treatment if it appeared to be significant (p<0.05) in the full model.

All analyses were repeated for each subgroup of patients treated with duloxetine and SSRIs to examine whether the association between depression-related pain (SSI-pain score) and functional impairment (SDS total score) was mediated or moderated by the type of treatment. In addition, the MMRM analysis was further conducted to compare the impacts of duloxetine and SSRIs on depression-related pain during follow-up. The same list of independent variables aforementioned were considered in the model.

All statistical analyses were performed using SAS version 9.3 for Windows (SAS Institute, Cary, NC, USA).

## 3 RESULTS

### 3.1 The Characteristics of Patients at Baseline

A total of 275 patients were included in the final analysis. Overall, the mean (standard deviation [SD]) age of these patients was 37.3 (SD=8.4) years, and only about one-third of them (34.6%) were female (27.1% in Saudi Arabia and 44.2% in the UAE, p=0.003). Slightly more than half of the patients (56.4%) were from Saudi Arabia, and the rest were from the UAE (43.6%). The majority of the patients (76.7%) were living with a spouse. About two-thirds of the patients (67.3%) had full-time employment, and more than half of the patients (57.8%) completed at least a tertiary or university degree. In addition, nearly two-thirds of these MDD patients (61.5%) also suffered from PPS at baseline. Only 20% of MDD patients had taken antidepressants, and 48.4% (21.1% for benzodiazepines and the 27.3% for other medications) had taken other psychiatric medications in the previous 24 months.

Of the 275 patients, 152 initiated duloxetine at baseline and the rest (n=123) initiated an SSRI antidepressant at baseline. The most common SSRIs prescribed at baseline were escitalopram (38.2%), paroxetine (27.6%), and sertraline (20.3%). The median daily doses (interquartile ranges [IQRs]) of these antidepressants at baseline were 10.0mg/d (IQR: 10.0mg/d – 10.0mg/d) for escitalopram, 22.5mg/d (IQR: 20.0mg/d – 25.0mg/d) for paroxetine, 50.0mg/d (IQR: 50.0mg/d – 100.0mg/d) for sertraline, and 60.0mg/d (60.0mg/d – 60.0mg/d) for duloxetine.

Table (**1**) summarises the baseline patient characteristics by treatment cohorts. A higher proportion of the SSRI cohort were female (p=0.015) and from the UAE (p=0.003), whereas a higher proportion of the duloxetine cohort had completed a tertiary or university degree (p=0.013). In addition, a higher proportion of the SSRI cohort had taken other psychiatric medications in the previous 24 months (37.4% vs. 19.1%), whereas a higher proportion of the duloxetine cohort had taken benzodiazepines in the previous 24 months (26.3% vs. 14.6%) (p=0.001). Nevertheless, the levels of depression severity and depression-related pain at baseline were similar for the two cohorts, and the proportion of patients exhibiting PPS at baseline was also similar for the two (63.8% in the duloxetine cohort and 58.5% in the SSRI cohort, p=0.371).

### 3.2 The Level of Functional Impairment over Time by Treatment Cohort

Table (**2**) demonstrates the mean levels of functional impairment at baseline and at 24 weeks, respectively, by treatment cohort. At baseline, the level of functional impairment was slightly higher in the duloxetine cohort than in the SSRI cohort: the mean SDS total score at baseline was 17.36 (SD=6.00) in the former and 15.66 (SD=6.28) in the latter (p=0.012). Nevertheless, both treatment groups, on average, had a moderate level of functional impairment in all three subdomains (SDS work, social life, and family life), with a mean score of 5.35 (SD=2.66) (for work) to 6.24 (SD=2.16) (for social life) in the duloxetine cohort and 5.11 (SD=2.41) (for work) to 5.35 (SD=2.17) (for social life) in the SSRI cohort.

Despite a higher level of baseline functional impairment, the duloxetine cohort achieved a greater level of functioning at 24 weeks, compared to the SSRI cohort. At 24 weeks, the mean SDS total score was 3.41 (SD=3.06) in the former and 4.60 (SD=3.21) in the latter (p=0.008). Similar patterns were also observed in all three SDS subdomains. This treatment difference was equivalent to the effect size of -0.38 for SDS total (subdomains: -0.25 for SDS social life to -0.47 for SDS work).

**Fig. (1):**
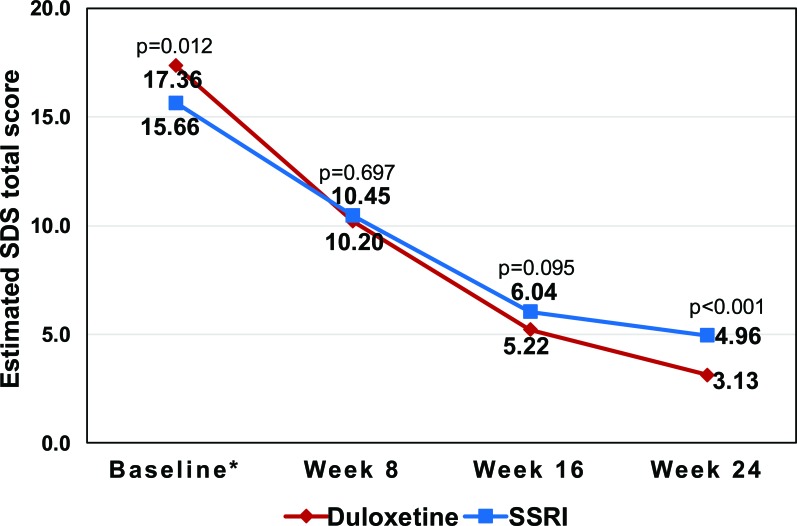
The estimated SDS total scores during follow-up, by treatment cohorts.

The level of functional impairment at 24 weeks was still lower in the duloxetine cohort than in the SSRI cohort even when the adjustment of baseline differences was made through the MMRM, as shown in Table (**3**) and Fig. (**1**). At 24 weeks, the estimated mean SDS total score (*i.e.*, least squares [LS] means) was 3.13 (standard error [SE]=0.26) in the former, which was lower than that of 4.96 (SE=0.29) in the latter (p<0.001) (*i.e.* better functioning in the duloxetine cohort) (Fig. **1**).

**Fig. (2):**
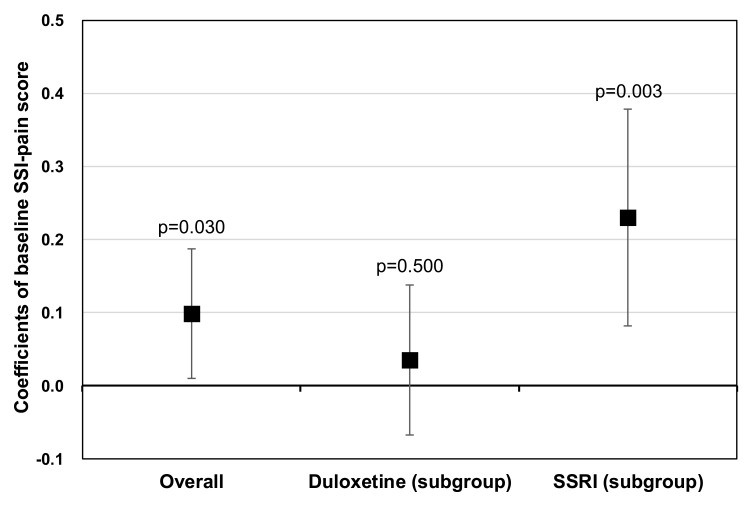
The results of MMRM analyses: associations between baseline SSI-pain scores and SDS total scores during follow-up, by treatment cohorts.

Notably, the level of depression-related pain at baseline, as measured by SSI-pain scores, was also statistically significantly associated with the level of functional impairment during follow-up (coefficient=0.10, p=0.030). This positive association between the two was even more apparent in the subgroup of SSRI-treated patients (coefficient=0.23, p=0.003), but not in the subgroup of duloxetine-treated patients (coefficient=0.04, p=0.500) (Fig. **2**). In relation to this finding, an additional analysis was conducted to confirm the differential impacts of duloxetine and SSRIs on depression-related pain. The MMRM results showed that the level of depression-related pain at 24 weeks was statistically significantly lower in duloxetine-treated patients (LS means [SE]: 8.31 [0.24]) than in SSRI-treated patients (LS means [SE]: 11.14 [0.28]) (p<0.001) (data not shown).

## 4 DISCUSSION

This *post-hoc* analysis assessed the level of functional impairment over time, using the Sheehan Disability Scale [32], in the subgroup of Middle Eastern patients with MDD, who were either treated with duloxetine or with an SSRI for up to 6 months in actual clinical practice in Saudi Arabia and the UAE. The results showed that this subgroup of patients had a moderate level of functional impairment in all three SDS subdomains (work, social life, and family life) at baseline, but the patients, particularly those patients treated with duloxetine, achieved better functioning over time in all three subdomains. The findings also highlighted the differential impacts of depression-related pain on functioning by treatment cohorts. The negative impact of baseline pain severity on improvement in functional impairment during follow-up appeared to be moderated by treatment with duloxetine, but not by treatment with SSRIs.

### 4.1 Effects and Roles of Antidepressants in the Treatment of MDD in The Middle East

Treatment with antidepressants, both duloxetine and SSRIs, was found to be effective in improving functioning of MDD patients in the Middle East. These findings are highly consistent with those of our previous studies with East Asian patients, which examined the effects of duloxetine with a daily dose of ≤ 60mg and SSRIs, respectively, on various clinical and humanistic outcomes [24, 26]. There are, however, no studies in the Middle East to have evaluated the effects of antidepressants in the treatment of MDD, as confirmed in the recent systematic review by Travers *et al.* [21]. This is rather surprising given the role of antidepressants in modern psychiatry and the high level of MDD burden (in terms of DALYs) reported in this region [3, 4].

No regional treatment guidelines for MDD are available either, except for the two published in a neighbouring country, Turkey [35, 37]. While both Turkish and other international guidelines [38, 39] recommend the use of antidepressants as first-line treatment of MDD, available evidence suggests that the rate of antidepressant use is low, but that of benzodiazepine is relatively high in the treatment of MDD in the Middle East [21]. In our study, 21.1% and 20.0% of the MDD patients were reported to have taken benzodiazepines and antidepressants, respectively, in the 24 months prior to the study entry. The lack of regional clinical evidence might have contributed in part to the absence of treatment guidelines and the lack of appropriate treatments for MDD in this region.

While our study found both duloxetine and SSRIs to be effective in improving functional impairment, duloxetine-treated patients achieved a higher level of functioning than SSRI-treated patients during follow-up. However, the superiority of SNRIs including duloxetine over SSRIs has not been well established in the literature [40], although limited evidence supports the advantage of SNRIs in subgroups of MDD patients with PPS and/or more severely ill [18, 41]. Notably, the majority of MDD Middle Eastern patients in this study exhibited PPS at baseline (63.8% in the duloxetine cohort and 58.5% in the SSRI cohort, p=0.371).

### 4.2 Implications of Depression-Related Pain in the Treatment of MDD in the Middle East

Our study also found differential impacts of depression-related pain on functioning by treatment cohorts. Duloxetine-treated patients achieved functioning independently of pain severity, but SSRI-related patients did not. That is, SSRI-treated patients who had a higher level of depression-related pain at baseline did not achieve the same level of functioning as those who had a lower level of depression-related pain at baseline, confirming the negative impact of pain on improvement in functioning in this cohort. This finding adds to the existing evidence that supports the advantages of SNRIs in the subgroup of patients with PPS, as discussed above, and also reaffirms the importance of pain control in the treatment of MDD.

Painful physical symptoms seemingly play dual roles in the treatment of MDD as both a trigger for help-seeking and a risk factor for delayed diagnosis. Its dual role is particularly relevant in the Middle East, where mental illness carries an intense and enduring stigma [42]. Patients in such cultures often tend to somatise their emotional problems and express them in terms of physical symptoms. This may partly explain the high percentage of painful physical symptoms (61.5% at baseline) found in our study, although it is not clear whether this is a manifestation of depressive symptoms or a coexisting pain condition with an unknown aetiology.

Okasha reported that up to 80% of psychiatric patients have a tendency to somatise their emotional and psychiatric problems [43]. Many of these patients therefore do not seek help in a psychiatric setting, but instead present to primary care with physical complaints. This may explain the over-representation of depressed patients in primary care in the Middle East. Using the Beck Depression Inventory, Hamdan *et al.* [12] found that nearly half of women attending primary care clinics (n=224) in the UAE were mildly (14.3%), moderately (14.7%), or severely (17.9%) depressed (12). Similarly, Becker *et al.* reported that about one in five patients attending primary care clinics (n=431) in Saudi Arabia had depression and 67.4% of them were female, using the Patient Health Questionnaire (PHQ) [44]. The authors also suggested that the restrictive Arab culture may have placed women at higher risk for somatic presentations of psychological distress in primary care settings. This postulation is in line with the low proportion of women (34.6%) included in our study, which was conducted in an outpatient psychiatric setting in the region. Becker further examined whether there is a discrepancy in the detection rates of depression and somatisation between primary care physicians and the use of the PHQ screening instrument [45]. The study reported that primary care physicians were aware of psychiatric disorders, but their diagnostic skills for these conditions were poor.

Taken together, a substantial proportion of MDD patients in the Middle East also suffer from painful physical symptoms, which are likely to negatively impact on clinical outcomes, including functioning. Many of these patients tend to seek help first from primary care physicians. It is therefore important to make sure that primary care physicians, who are at the front-line of the healthcare system, correctly diagnose and treat MDD even in the presence of painful physical symptoms.

### 4.3 Study Limitations

While this study provides meaningful clinical insights into MDD in the Middle East, its findings should be interpreted in the context of the following study limitations. Firstly, data were taken from an observational study. Although the MMRM analysis adjusted for the baseline imbalance between duloxetine- and SSRI-treated patients, there still exists the unobserved imbalance between the two cohorts. Secondly, the primary objective of the observational study was to assess TESD in the treatment of MDD. The study, therefore, included only those patients who were sexually active and did not have sexual dysfunction at baseline. It has been reported that sexual dysfunction is two to three times more prevalent in patients with depression than in the general population [46, 47]. Our findings may therefore not be immediately generalizable to MDD patients as a whole. In addition, sexual dysfunction was measured using the Arizona Sexual Experience Scale [30]. It is not clear whether patients, especially women, were willing to accurately report their sexual dysfunction, particularly in this restrictive culture. Further research is needed to examine whether our findings can be replicated in MDD patients without such inclusion/exclusion criteria. Thirdly, even within the SSRI class, there exist differences in efficacy and/or tolerability between the individual drugs [48]. Therefore, our findings cannot represent the differences in effectiveness, in terms of functional improvement, between duloxetine and any specific SSRIs. Finally, this *post-hoc* analysis included only 275 patients from the Middle East, and it may therefore not be representative of all patients with MDD in the region. Our results should be interpreted with caution until further replication is available.

## CONCLUSION

Despite its limitations, this study provides some useful clinical insights into the treatment of MDD in the Middle East. Patients treated with duloxetine achieved higher levels of functioning (*i.e.*, global, work, social life, and family life), compared to those treated with SSRIs for the management of MDD in actual clinical settings in the Middle East (Saudi Arabia and the UAE). The study also revealed a high proportion of MDD patients (61.5%) suffering from painful physical symptoms, which was found to negatively impact on functioning. The negative impact of depression-related pain was, however, moderated by treatment with duloxetine, but not by treatment with SSRIs. These findings add to the existing but limited evidence that supports the additional advantage of duloxetine, and possibly SNRIs as a whole, over SSRIs for the treatment of MDD patients presenting with painful physical symptoms. Given the high level of PPS among Middle Eastern MDD patients, it is particularly important to make sure that local primary care physicians, who are at the front-line of the healthcare system, appropriately diagnose and treat MDD patients presenting with physical complaints.

## Figures and Tables

**Table 1:** Baseline patient characteristics, by treatment cohorts.

**Baseline Characteristic**	**Duloxetine** **(*n*=152)**	**SSRI (*n*=123)**	**P-value**
Age, Mean (SD)	36.9 (7.9)	37.7 (9.1)	0.673
Female, %	28.3	42.3	0.015
Caucasian, %	83.6	86.2	0.547
United Arab Emirates, %	35.5	53.7	0.003
Age at First Symptoms of MDD, Mean (SD)	31.5 (7.3)	31.3 (8.6)	0.787
BMI (kg/m^^2^^), Mean (SD)	26.9 (4.2)	26.5 (4.1)	0.582
Living with a Spouse, %	77.0	76.4	0.838
Independent Living, %	13.8	15.4	0.703
Level of Education, %	-	-	0.013
University or More	63.8	50.4	-
Secondary School/Occupational Programme	32.2	37.4	-
Primary School or Less	3.9	12.2	-
CGI-S, Mean (SD)	4.5 (0.6)	4.5 (0.6)	0.310
QIDS-SR_16_, Mean (SD)	12.6 (4.5)	12.0 (4.9)	0.234
SSI-pain, Mean (SD)	15.7 (4.9)	14.7 (4.9)	0.098
Painful Physical Symptoms, %	63.8	58.5	0.371
Number of Comorbidities, %	-	-	0.050
None	87.4	76.0	-
1	11.3	21.5	-
≥ 2	1.3	2.5	-
Had MDD Episodes in the Past 24 Months, %	78.9	82.1	0.511
Had been Hospitalised for MDD in the Past 24 Months, %	2.6	6.5	0.118
Had Taken Antidepressants in the Past 24 Months, %	21.1	18.7	0.885
Had Taken other Psychiatric Medications in the Past 24 Months, %	-	-	0.001
Benzodiazepines	26.3	14.6	-
Other	19.1	37.4	-
None	54.6	48.0	-

**Abbreviations:** BMI, Body Mass Index; CGI-S, Clinical Global Impressions of Severity; MDD, Major Depressive Disorder; QIDS-SR_16_, 16-item Quick Inventory of Depressive Symptomatology Self-Report; SD, Standard Deviation; SSI, Somatic Symptom Inventory; SSRI, Selective Serotonin Reuptake Inhibitor

**Table 2:** Mean levels (raw values) of SDS total and subscores at baseline and at 24 weeks, by treatment cohorts.

**Outcome**	**Duloxetine**	**SSRI**	**P-value**	**Effect size** ^a^
**At Baseline**				
**SDS Total Score**	**17.36 (6.00)**	**15.66 (6.28)**	**0.012**	**0.28**
SDS Work Score	5.35 (2.66)	5.11 (2.41)	0.186	0.09
SDS Social Life Score	6.24 (2.16)	5.35 (2.17)	<0.001	0.41
SDS Family Life Score	5.77 (2.29)	5.20 (2.19)	0.019	0.25
**At 24 weeks**				
**SDS Total Score**	**3.41 (3.06)**	**4.60 (3.21)**	**0.008**	**-0.38**
SDS Work Score	1.09 (1.23)	1.67 (1.27)	0.001	-0.47
SDS Social Life Score	1.24 (1.04)	1.51 (1.11)	0.087	-0.25
SDS Family Life Score	1.08 (1.01)	1.41 (1.04)	0.024	-0.32

^a^ These show effect sizes of differences in functional impairment between treatment cohorts. **Abbreviations:** SDS, Sheehan Disability Scale; SSRI, Selective Serotonin Reuptake Inhibitor

**Table 3:** The results of MMRM analyses: Factors associated with SDS total scores during follow-up.

**Parameter**	**Parameter** **Estimate**	**Standard** **Error**	**P-Value**
Intercept	9.35	1.67	<0.001
Age	-0.02	0.02	0.338
Female (vs. male)	-0.31	0.39	0.421
CGI	-0.18	0.39	0.642
QIDS-SR_16_ Score at Baseline	-0.31	0.06	<0.001
SSI-Pain Score at Baseline	0.10	0.05	0.030
SDS Total Score at Baseline	0.31	0.03	<0.001
Duloxetine (vs. SSRI)^a^	-0.25	0.64	0.697
Weeks (vs. Week 8)^a^			
Week 16	-4.41	0.44	<0.001
Week 24	-5.49	0.48	<0.001
Weeks*Treatment^a^			
Duloxetine at Week 16	-0.58	0.58	0.324
Duloxetine at Week 24	-1.58	0.65	0.015

^a^ As the model included the interaction term between treatment and time (weeks), the interpretation of the coefficients of treatment and time is not straightforward. The least squares mean of the SDS total score at each post-baseline visit by treatment cohorts was further estimated and presented in (Fig. **1**). **Abbreviations:** QIDS-SR_16_, 16-item Quick Inventory of Depressive Symptomatology Self-Report; SDS, Sheehan Disability Scale; SSI, Somatic Symptom Inventory; SSRI, Selective Serotonin Reuptake Inhibitor
